# Maternal DCAF13 Regulates Chromatin Tightness to Contribute to Embryonic Development

**DOI:** 10.1038/s41598-019-42179-w

**Published:** 2019-04-18

**Authors:** Yang Liu, Long-Wen Zhao, Jing-Ling Shen, Heng-Yu Fan, Yan Jin

**Affiliations:** 1 Laboratory of Medical Genetics, Harbin Medical University, Harbin, China; 2 Life Sciences Institute, Zhejiang University, Hangzhou, 310058 China

**Keywords:** Embryogenesis, Oogenesis

## Abstract

Maternal-zygotic transition (MZT) is critical for the developmental control handed from maternal products to newly synthesized zygotic genome in the earliest stage of embryogenesis. However, the spatiotemporal dynamic regulation of MZT by maternal factors is largely unknown. Here, we reported a novel maternal factor, DCAF13, which was highly expressed in growing oocyte nucleolus and had key maternal effects on oocyte and zygotic chromatin tightness during maternal to zygotic transition. DCAF13 specifically deleted in oocytes resulted in loose chromatin structure in fully grown germinal vesicle oocytes. Despite normal nuclear maturation in maternal DCAF13-deleted oocytes, the chromosomes at MII stage were not properly condensed. Consequently, the nuclear and nucleolar structure reorganized abnormally, and transcription was inactive in zygotic embryos. RNA-seq analysis of MII oocytes and 2-cell embryos demonstrated that the transcriptomes between knockout and control oocyte were similar, but the maternal DCAF13 deleted two-cell embryos showed a significant decrease in transcription. In addition, the maternal DCAF13-deleted embryos displayed arrest at the two-cell stage, which could not be rescued by injecting *flag-Dcaf1*3 mRNA in the zygote. This revealed that DCAF13 was a unique maternal effect factor regulating the nucleolus.

## Introduction

In vertebrates, maternal-zygotic transition (MZT) is not a one-stage process, but a process that is active from fertilization to the step when the zygotic genome is fully activated, and maternal mRNAs are completely degraded^1^. Eventually, successful zygotic genome activation (ZGA) is required for further embryo development, failure of which causes growth arrest. Prior to zygotic genome activation, the initiation of zygotic development and nuclear reorganization are under the control of maternal factors^2,3^, which occurs at the late two-cell stage in mice and four–eight-cell stage in humans^4^.

The morphology and structure of the nucleus need to be reformed to adjust to the transcription during MZT^5^. During the period of oocyte growing, the chromatin is less condensed and not confined around the nucleolus, termed the non-surrounded nucleolus (NSN) configuration^6^. However, when it comes to fully grown oocytes, there is a shutdown in new transcript synthesis, and the chromatin is rather condensed and particularly confined around the nucleolus, termed the surrounded nucleolus (SN) configuration^7^. Although the chromatin is condensed during meiosis, once fertilization, the chromatin structure of pronucleus (PN) in zygote turns to be extremely loose, leading to the whole genome to be poorly transcribed in the zygote^8^. However, the detailed regulatory mechanism behind this is largely unclear.

The maternal nucleus structure or factors affect ZGA^9^. Embryos derived from denucleated oocytes fail to develop beyond the two-cell stage, suggesting that the oocyte nucleus has a pivotal role in early embryonic development^10^. These studies suggest that the nucleus/nucleolus morphology and chromatin configuration need to be changed to adapt to the gradually recovered transcription during MZT.

DCAF13 is one of the major substrate adaptors of the DDB1/CUL4 complex, which is an E3 ligase^11^. In our previous studies, *Dcaf13* knockout embryos are arrested at the morula stage, accompanied with increased H3K9me3 level in blastomeres of 8-cell embryos. Furthermore, we show that DCAF13 bridges ubiquitin E3 ligase CRL4 to histone methyltransferase SUV39H1, and target this enzyme for polyubiquitination and proteasomal degradation. As a result, CRL4-DCAF13 facilitates H3K9me3 removal and zygotic gene expression in preimplantation embryos^12^. In order to explore the role of maternal DCAF13 in oocytes, we specifically deleted *Dcaf13* in developing oocytes, as early as the primordial follicle stage using *Cre* transgene driven by *Gdf9* promoter (*Gdf9-Cre)*. In this mouse model, the majority of the oocytes fail to develop to the preovulatory stage and became apoptotic. The female mice are infertile due to premature ovarian failure. Mechanistically, DCAF13 binds with the nucleolar protein fibrillarin and is involved in the processing of 18S rRNA during oocyte growth^13^.

However, the function of maternal DCAF13 in MZT is still largely unclear. In this study, we generated conditional knockout mice with a deletion of *Dcaf13* in oocytes from primary follicles using *Zp3-Cre*, which was a powerful model to elucidate the function of maternal DCAF13 during the early development of the zygotes^14^. We found that maternal DCAF13 participated in the zygotic nucleus/nucleolus reformation and function and was involved in the switch of chromatin tightness during MZT. By DCAF13 maternally depletion, the zygote cannot activate its genome transcription, render the embryo development, and arrest at two-cell stage. Together, our results pointed out DCAF13 as a new maternal factor that regulated chromatin tightness and affected the zygotic nucleus/nucleolus reformation and ZGA.

## Results

### DCAF13 is essential for female fertility but not for oocyte meiotic maturation

**Figure 1:**
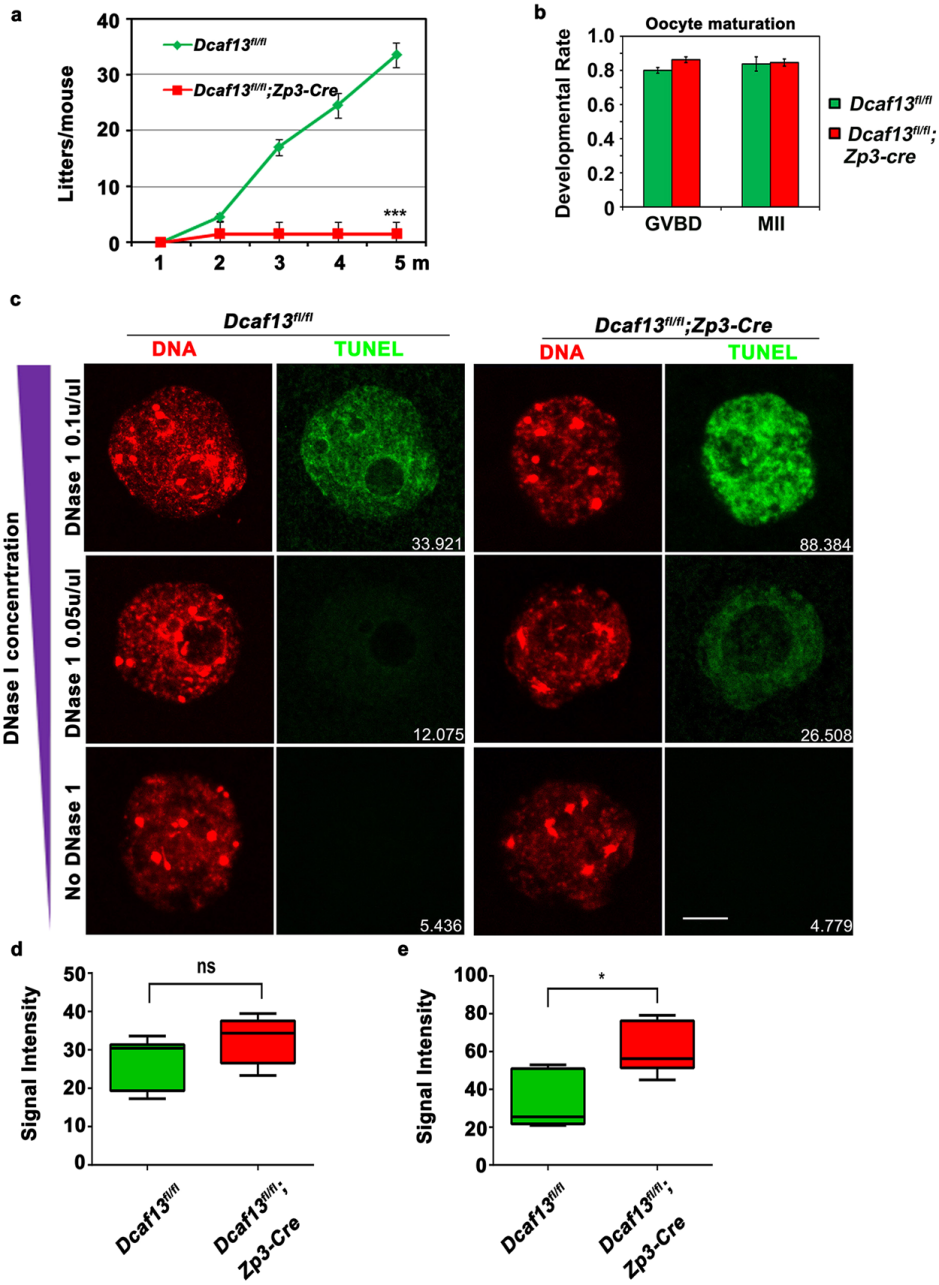
DCAF13 is essential for female fertility but not for oocyte meiotic maturation, and deletion of DCAF13 results in loose chromatin structure in fully grown germinal vesicle (GV) oocytes. (**a**) Cumulative numbers of pups per female (*Dcaf13*^*fl*/*fl*^ and *Dcaf13*^*fl*/*fl*^*;Zp3-Cre*) (n ≥ 5). Error bars indicated standard error of the mean (SEM). *** to *p* < 0.001. (**b**) Rates of GVBD and PB emission of *Dcaf13*^*fl*/*fl*^ and *Dcaf13*^*fl*/*fl*^*;Zp3-Cre* oocytes cultured *in vitro*. Scale bar, 100 µm. *Dcaf13*^*fl*/*fl*^ (n = 75), *Dcaf13*^*fl*/*fl*^*;Zp3-Cre* (n = 50). (**c**) Lack of DCAF13 in oocytes led to increased DNase I accessibility. Scale bar, 25 µm. (**d**,**e**) The positive TUNEL signal of 0.05 U/µL and 0.1 U/µL DNase I is indicated. * to *p* < 0.05, ns to *p* > 0.05. 0.1 U/µL DNase I: *Dcaf13*^*fl*/*fl*^ (n = 10), *Dcaf13*^*fl*/*fl*^*;Zp3-Cre* (n = 12). 0.05 U/µL *Dcaf13*^*fl*/*fl*^ (n = 12), *Dcaf13*^*fl*/*fl*^*;Zp3-Cre* (n = 12).

In mouse oocytes, DCAF13 expression decreases during the transition from mature to climacteric stage. In a previous study, we found that DCAF13 was localized in the nucleolus and was essential for 18S rRNA transcription and nucleolus function in growing oocytes^13^. However, whether DCAF13 is essential for female fertility is unclear. We used the growing oocyte-specific deletion strategy by crossing *Zp3-Cre* transgenic mice with the *Dcaf13*^*fl*/*fl*^ mice, in which the *Dcaf13* gene was deleted specifically in oocytes from the primary follicle stage. *Dcaf13*^*fl*/*fl*^;*Zp3-Cre* (*Dcaf13*^*oo*−/−^) females were then mated with wild-type males. Strikingly, we found that progeny was barely obtained compared to *Dcaf13*^*fl*/*fl*^ females. This indicated that *Dcaf13*^*fl*/*fl*^;*Zp3-Cre* female were sterile (Fig. 1a). To determine whether the reasonof sterility was the defect in oocytes, we isolated fully grown GV stage oocytes from *Dcaf13*^*fl*/*fl*^ and *Dcaf13*^*fl*/*fl*^;*Zp3-Cre* females and investigated the *in vitro* oocyte meiotic maturation process. We found that the *Dcaf13*^*fl*/*fl*^;*Zp3-Cre* oocytes did not show any defect in GVBD or PB1 emission (Fig. 1b). To further confirm the quality of oocytes, we also detected several rRNA level (18S rRNA, 28S rRNA, and 5.8S rRNA) which were the products of pre-rRNA processing. The results showed that rRNA processing in oocytes was not significantly affected in these fully-grown oocytes collected from young females of *Dcaf13*^*fl*/*fl*^;*Zp3-Cre* mice (see Supplementary Fig. S2).

### DCAF13 deletion results in loose chromatin structure in fully grown GV oocytes

At the fully grown GV stage, the transcription of the oocytes was inactive, and the chromatin appeared as a condensed perinucleolar ring (SN oocytes). However, in *Dcaf13*^*fl*/*fl*^;*Zp3-Cre* GV stage oocytes, the SN oocytes were notably less than those in *Dcaf13*^*fl*/*fl*^ GV stage oocytes, and most of their chromatin was dispersed (NSN oocytes), indicating that DCAF13 regulated the chromatin configuration. To confirm the hypothesis of DCAF13 function in the regulation of chromatin configuration, we performed a DNase I sensitivity assay to test the tightness of chromatin. If the chromatin structure was loose, it was easily exposed to DNase I and would show higher sensitivity. As we predicted, when the DNase I concentration increased, *Dcaf13*^*fl*/*fl*^;*Zp3-Cre* oocytes showed clear signals of DNA damage by increased levels of terminal deoxynucleotidyl transferase (TdT)-mediated deoxyuridine triphosphate (dUTP) nick-end labeling (Fig. 1c–e). Therefore, we concluded that the chromatin tightness level decreased in DCAF13-deleted oocytes.

### DCAF13-maternally depleted embryos display abnormal chromatin tightness level and nuclear configuration

**Figure 2:**
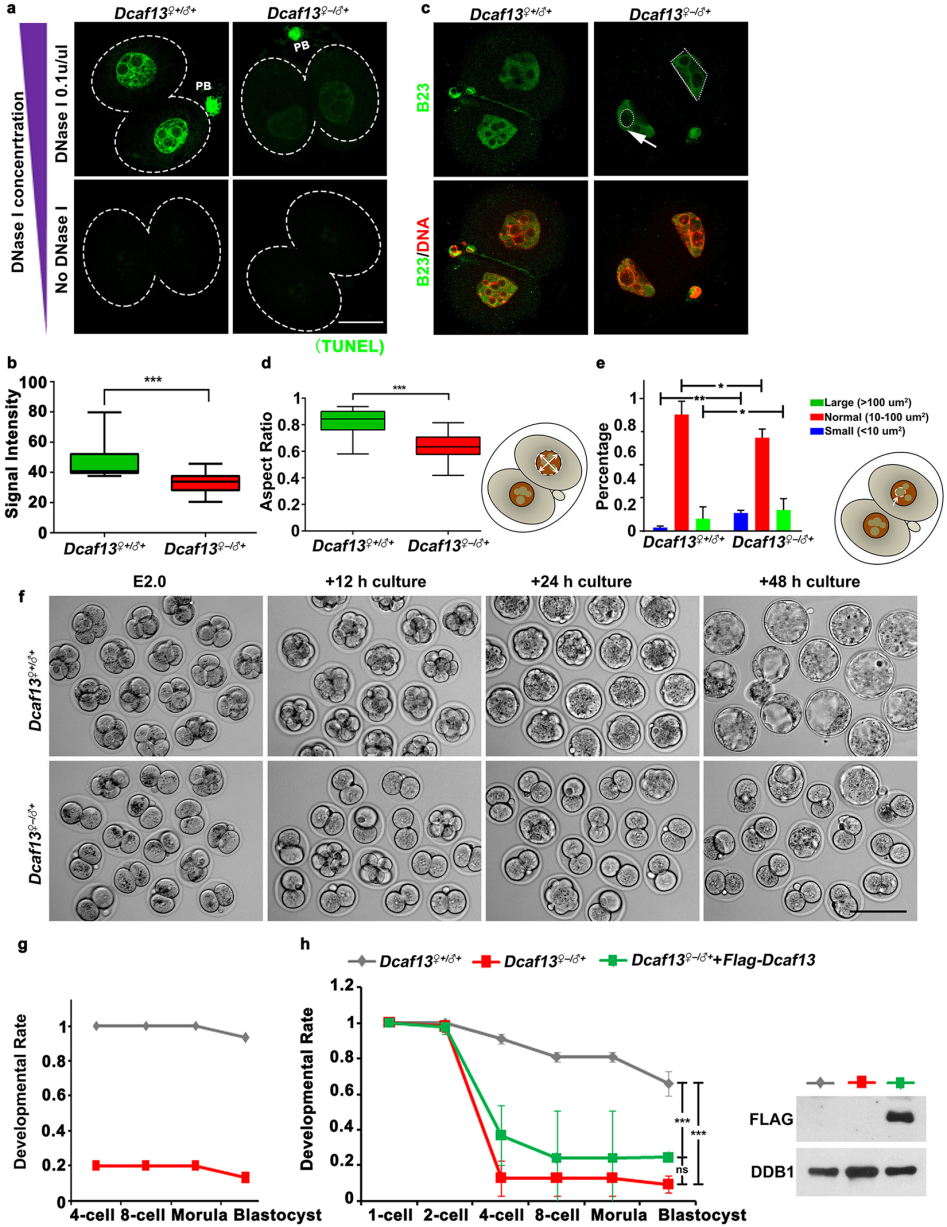
DCAF13-maternally depleted embryos have abnormal chromatin tightness levels and nuclear configuration, and are arrested at the two-cell stage. (**a**) DCAF13-maternally depleted embryos showed decreased DNase I accessibility. Scale bar, 20 µm. (**b**) The positive TUNEL signal of 0.1 U/µL was indicated. *** to *p* < 0.001. *Dcaf13*^*♀*+/*♂*+^ (n = 12), *Dcaf13*^*♀*−/*♂*+^ (n = 20). (**c**) Immunofluorescence using antibodies against B23 (in green) and DNA (in red) in two-cell embryos. The abnormal nuclear morphology (white dotted line) and the abnormal nucleoli (white arrow) were indicated. Scale bar, 20 µm. Abnormal nuclear morphology was quantified at (**d**) Classification of nucleoli at (**e**). *p < 0.05, **p < 0.01. (**f**) Representative images for two consecutive days of *in vitro* culture for *Dcaf13*^*♀*+/*♂*+^ or *Dcaf13*^*♀*−/*♂*+^ embryos collected at E2. Scale bar, 100 µm. (**g**) Rates of embryo development of (**f**). (**h**) The numbers of zygotes, two-cell, four-cell, eight-cell, morula, and blastocysts were counted at each time point (left). Western blot results showed levels of FLAG-DCAF13 in 2-cell embryos with or without mRNA microinjection at the zygote stage. DDB1 was blotted as a loading control (right). ns to *p* > 0.05, *** to *p* < 0.001. *Dcaf13*^*♀*+/*♂*+^ (n = 64), *Dcaf13*^*♀*−/*♂*+^ (n = 71), *Dcaf13*^*♀*−/*♂*+^ with microinjection of FLAG-tagged DCAF13 (n = 57).

Owing to the chromatin configuration defects observed following the deletion of DCAF13 in oocytes, we next sought to study whether this defect also perturbed the reorganization of the nucleus and nucleolus after fertilization. First, we investigated the chromatin tightness of the WT and *Dcaf13*^*♀*−/*♂*+^ embryos. With the increased concentration of DNase I, we examined the signals of DNA damage and quantified the relative fluorescence intensity. Upon treatment with 0.1 U/µL DNase I, *Dcaf13*^*♀*−/*♂*+^ two-cell embryos showed less DNA damage compared to control two-cell embryos (Fig. 2a,b).

To address the interconnection between nuclear morphology and chromatin tightness, we investigated the nuclear morphology of them. We found that abnormal nuclear and nucleolus morphology was observed in *Dcaf13*^*♀*−/*♂*+^ two-cell embryos (Fig. 2c). To measure this change, we set up a method to measure the ratio of minor-major axis. In *Dcaf13*^*♀*+/*♂*+^ two-cell embryos, the nucleus was close to roundness, and the ratio was close to 1. But in *Dcaf13*^*♀*−/*♂*+^ two-cell embryos, the nucleus morphology was abnormal, and the ratio was deviated to 1 (Fig. 2d). The most striking effect we detected is the morphology of the nucleoli. We found that the size of the nucleoli was not uniform in *Dcaf13*^*♀*−/*♂*+^ two-cell embryos. By classifying the nucleoli based on area scores, the number and percentage of nucleoli which had abnormal size was elevated in *Dcaf13*^*♀*−/*♂*+^ two-cell embryos (Fig. 2e). In both, chromatin tightness levels were abnormally elevated and nucleus reorganization was failed in DCAF13-maternally depleted two-cell embryos.

### Depletion of maternal DCAF13 results in developmental arrest at the two-cell stage

Next, we mated *Dcaf13*^*fl*/*fl*^ and *Dcaf13*^*fl*/*fl*^;*Zp3-Cre* females with wild-type males, and then embryos were isolated on embryonic day 2 (E2) (Fig. 2f). Surprisingly, we found that most of the *Dcaf13*^*♀*−/*♂*+^ embryos were arrested at the two-cell stage, and the proportion of *Dcaf13*^*♀*−/*♂*+^ four-cell stage embryos recovered (~20%) was notably lower than that obtained with *Dcaf13*^*♀*+/*♂*+^ embryos (100%) (Fig. 2g). To assess the developmental fate of *Dcaf13*^*♀*−/*♂*+^ two-cell embryos and surviving *Dcaf13*^*♀*−/*♂*+^ four-cell embryos, we kept culturing and monitoring them *in vitro*. After 48 h in culture, all the surviving *Dcaf13*^*♀*−/*♂*+^embryos were found to be at the blastocyst stage, like *Dcaf13*^*♀*+/*♂*+^ embryos, but two-cell embryos were confirmed arrested not developmental delayed (Fig. 2f,g).

To determine if the embryo arrest at the two-cell stage was caused by maternal DCAF13 deletion, we compensated DCAF13 in *Dcaf13*^*♀*−/*♂*+^ zygotes by injecting *Flag-Dcaf13* mRNA. As shown in Fig. 2h, 75.5% embryos compensated with DCAF13 (in *Dcaf13*^*♀*−/*♂*+^) zygotes were found to be significantly blocked at the two-cell stage compared to 9% in *Dcaf13*^*♀*+/*♂*+^ embryos. Minor rescues were found with *Flag-Dcaf13* mRNA injection for *Dcaf13*^*♀*−/*♂*+^ embryos arrested at the two-cell stage, although exogenous FLAG-DCAF13 was effectively expressed at the 2-cell stage (Fig. 2h). Uncropped scans of the Western blot results of DCAF13 expression was provided in Supplementary Fig. S1. Taken together, the maternal DCAF13 was required for zygotes to proceed beyond the two-cell stage.

### Maternal DCAF13 affects zygotic genome activation by the two-cell stage

**Figure 3:**
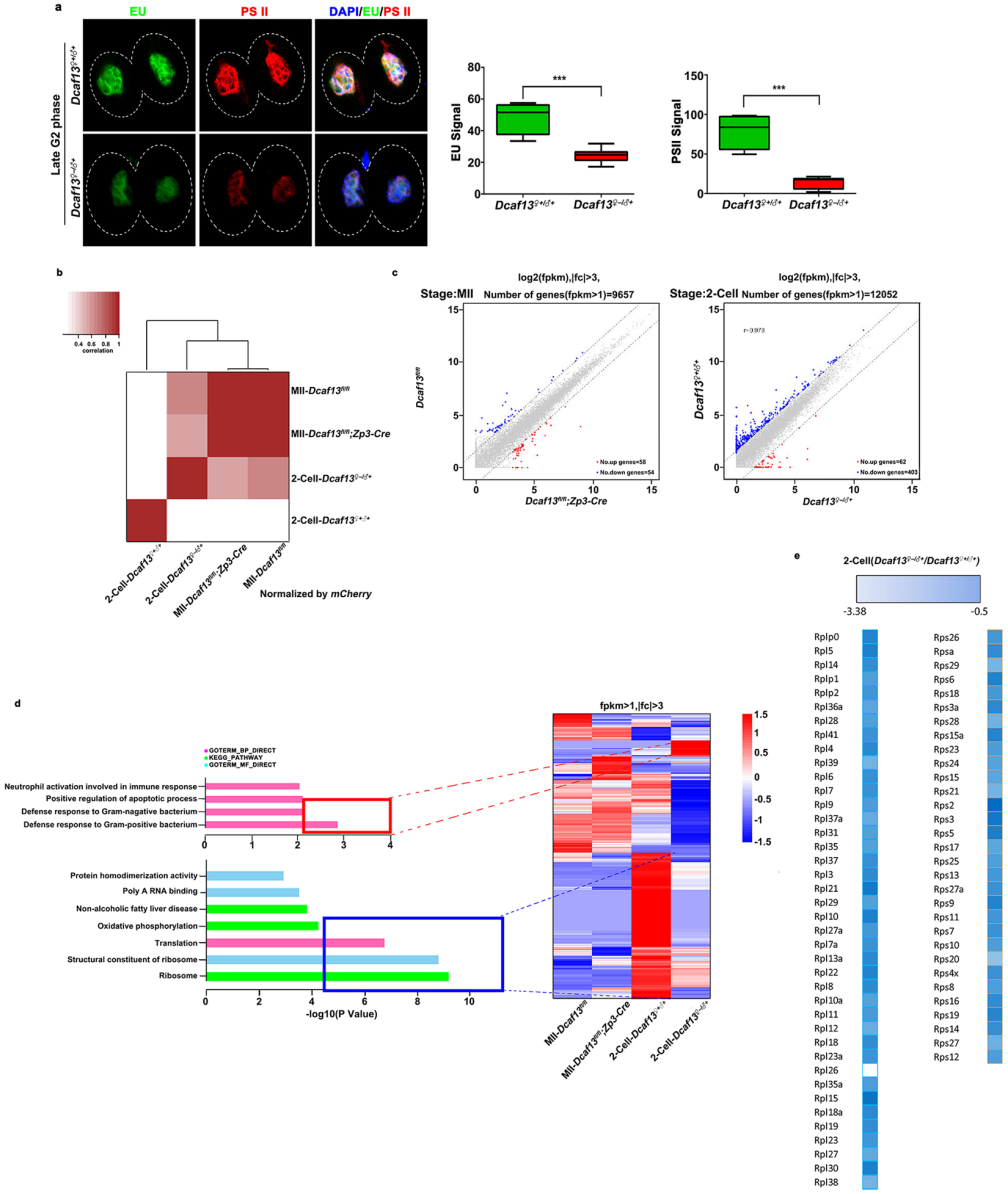
Maternal DCAF13 affects zygotic genome activation by the two-cell stage. (**a**) Detection of newly synthesized RNA by 5-ethynyl uridine (EU) (in green) incorporation and the activity of polymerase II (POLII) (PSII in red) in two-cell embryos of *Dcaf13*^*♀*+/*♂*+^ or *Dcaf13*^*♀*−/*♂*+^ is shown (top), and the measurement of the intensity was indicated (bottom). Scale bar, 20 µm. *** to *p* < 0.001. *Dcaf13*^*♀*+/*♂*+^ (n = 10), *Dcaf13*^*♀*−/*♂*+^ (n = 10). (**b**) Heat map and cluster tree represented the relative mRNA levels of total transcripts in *Dcaf13*^*fl*/*fl*^ and *Dcaf13*^*fl*/*fl*^*;Zp3-Cre* MII oocytes as well as *Dcaf13*^*♀*+/*♂*+^ or *Dcaf13*^*♀*−/*♂*+^ two-cell embryos. (**c**) Gene scatter plots showed that transcript levels increasing more than three folds in *Dcaf13*^*fl*/*fl*^*;Zp3-Cre* MII oocytes (*Dcaf13*^*fl*/*fl*^*;Zp3-Cre*/*Dcaf13*^*fl*/*fl*^ > *3*) were upregulated (red); Transcript levels decreasing more than three folds (*Dcaf13*^*fl*/*fl*^/*Dcaf13*^*fl*/*fl*^*;Zp3-Cre* > 3) were downregulated (blue) (top). Transcript levels increasing more than three folds in *Dcaf13*^*♀*−/*♂*+^ two-cell embryos were upregulated (red); transcript levels decreasing more than three folds in *Dcaf13*^*♀*−/*♂*+^ two-cell embryos were downregulated (blue) (bottom). (**d**) Gene ontology (GO) analysis plot and heat map indicated the function of transcripts whose levels increased (red) or decreased (blue) in *Dcaf13*^*♀*−/*♂*+^ two-cell embryos compared to control. (**e**) Heat map described the level change of transcripts encoding ribosomal proteins grouped by ribosome large and small subunit in *Dcaf13*^*♀*−/*♂*+^ two-cell embryos. The fold changes (*Dcaf13*^*♀*−/*♂*+^/*Dcaf13*^*♀*+/*♂*+^) were listed at the top of the boxes from white to blue (from large to small).

After fertilization, new transcripts need to be generated along with protein synthesis corresponding to ZGA^15^. This progress mainly appears in the late two-cell stage. Therefore, we chose fertilized 54-h embryos (late two-cell stage) to detect the transcription levels. We performed EU staining and immunofluorescence detection of RNA Polymerase II CTD phospho-Ser2 (PolIISer2P), a form of active transcription. Of note, significant differences were detected between *Dcaf13*^*♀*+/*♂*+^ and *Dcaf13*^*♀*−/*♂*+^ two-cell embryos, and transcription activity decreased by more than half in *Dcaf13*^*♀*−/*♂*+^ two-cell embryos (Fig. 3a). To assess the whole transcriptome changes affected by the lack of maternal DCAF13, we investigated the gene expression profiling by performing RNA sequencing (RNA-seq) for MII oocytes and embryos at the two-cell stage with two biological replicates in a cohort of control and mutant samples (n = 10 each). The cDNA libraries were set up, and replicates from control and mutant oocytes as well as embryos were used (Fig. 3b); we applied Illumina-based deep RNA sequencing on these samples. *mCherry* mRNA was incorporated in the samples as the reference. And the mapping ratio of the samples were normal (Table 1). We normalized the FPKM values of genes to the FPKM values of *mCherry*. According to the FPKM values of genes, we divided transcripts into five groups in MII/two-cell stage (Table 2). We found that DCAF13 deleted in oocytes did not affect MII oocyte transcriptome dramatically, but maternal DCAF13 depletion caused a decrease in global transcription levels at the two-cell stage (Fig. 3b). Our analysis revealed that 62 genes were upregulated, and 403 genes were downregulated in the *Dcaf13*^*♀*−/*♂*+^ two-cell embryos compared to the *Dcaf13*^*♀*+/*♂*+^ embryos when the gene scattered by fold changes above three. Furthermore, the analysis of MII oocyte transcriptomes also revealed that a few genes were misregulated in DCAF13-deleted oocytes compared to maternal DCAF13-depleted embryos (Fig. 3c). This underlined the specificity of the effects caused by maternal DCAF13 in two-cell embryos.

**Table 1:** The mapping ratio of the RNA-seq samples.

Sample	Mapping ratio
MII-*Dcaf13*^*fl/fl*^	88.00%
MII-*Dcaf13*^*fl/fl*^*;Zp3-Cre*	79.30%
2-cell-*Dcaf13*^*fl/fl*^	69.10%
2-cell-*Dcaf13*^*fl/fl*^*;Zp3-Cre*	70.30%

**Table 2:** Gene table at certain FPKM threshold.

Stage	FPKM	Number of genes	
		*Dcaf13* ^*fl/fl*^ *;Zp3-Cre*	*Dcaf13* ^*fl/fl*^
MII	FPKM < = 0.1	9583	9310
	0.1 < FPKM < = 1	3808	3899
	1 < FPKM < = 3	2187	2234
	3 < FPKM < = 5	1078	1111
	5 < FPKM	5857	5999
2-cell	FPKM < = 0.1	7045	7441
	0.1 < FPKM < = 1	4092	3686
	1 < FPKM < = 3	2710	2441
	3 < FPKM < = 5	1360	1301
	5 < FPKM	7306	7644

Gene ontology analysis of up- and downregulated genes at the two-cell stage suggested that the apoptotic process-related genes were upregulated mostly in DCAF13-maternally depleted two-cell embryos, and, surprisingly, ribosome and translation-related genes were downregulated mostly after maternal DCAF13 depletion (Fig. 3d). To further analyze the expression changes of genes encoding ribosomal proteins, we generated the heat map of these genes grouped by ribosome large and small subunits (Fig. 3e). Strikingly, all genes were shown to be downregulated. In line with our previous work, we concluded that maternal DCAF13 deficiency could disorganize the zygotic genome activation.

### Absence of maternal DCAF13 abrogates zygotic nucleus function in transcription and results in decreased translation

**Figure 4:**
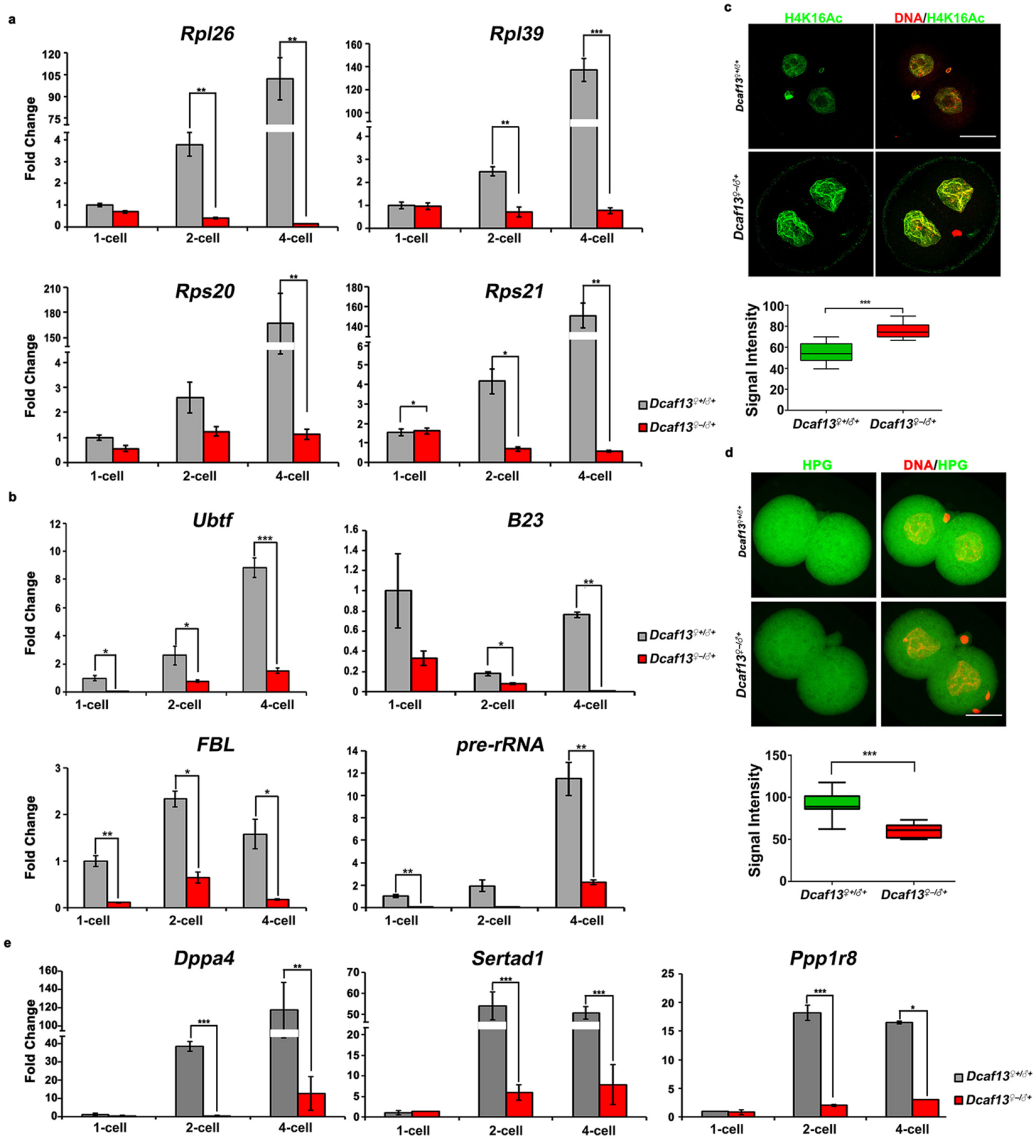
Absence of maternal DCAF13 abrogates zygotic nucleus function in transcription and results in decreased translation. (**a,b**) Graphical representation of the mean expression level ± standard error of the mean (SEM) by reverse transcription polymerase chain reaction (RT-PCR) for several ribosomal protein genes (**a**) and rDNA processing factor gene (**b**) in *Dcaf13*^*♀*+/*♂*+^ (in gray) or *Dcaf13*^*♀*−/*♂*+^ (in red) zygotes, two-cell and four-cell (two-cell stage in *Dcaf13*^*♀*−/*♂*+^) embryos. *p < 0.05, ** to *p* < 0.01 and*** to *p* < 0.001. (**c**) Immunofluorescence using antibodies against H4K16Ac (in green) and DNA (in red) in two-cell embryos (top), and intensity measurement (bottom). Scale bar, 20 µm. *** to *p* < 0.001. *Dcaf13*^*♀*+/*♂*+^ (n = 16), *Dcaf13*^*♀*−/*♂*+^ (n = 16). (**d**) Detection of newly synthesized protein by homopropargylglycine (HPG) incorporation in two-cell embryos of *Dcaf13*^*♀*+/*♂*+^ or *Dcaf13*^*♀*−/*♂*+^ was shown (top), also with the intensity measurement (bottom). Scale bar, 20 µm. *** to *p* < 0.001. *Dcaf13*^*♀*+/*♂*+^ (n = 14), *Dcaf13*^*♀*−/*♂*+^ (n = 14). (**e**) Graphical representation of the mean expression level ± SEM by RT-PCR for several representative zygotic genes in *Dcaf13*^*♀*+/*♂*+^ (in gray) or *Dcaf13*^*♀*−/*♂*+^ (in red) zygotes, two-cell and four-cell (two-cell stage in *Dcaf13*^*♀*−/*♂*+^) embryos. *p < 0.05, ** to *p* < 0.01, and *** to *p* < 0.001.

To further confirm the RNA-seq results, we selected several translation-related genes (*Rpl26, Rpl39, Rps20*, and *Rps21*) that should be transcript-activated, so that we could detect their transcription. Because the transcription of many genes were decreased after maternal DCAF13-depetion, exogenous *mCherry* mRNA was added to the samples as a control for normalization. Relative mRNA levels of other genes were compared to the levels of *mCherry* mRNA in the same sample. It could be concluded that all of the selected genes failed to be expressed at the two-cell stage in DCAF13-maternally depleted embryos compared to the control group (Fig. 4a). This phenotype was more remarkable when the control group developed to the four-cell stage.

Since the nucleolus morphology changed, and B23 staining intensity decreased in DCAF13-maternally depleted two-cell embryos, we hypothesized that the nucleolar function might be affected. The major function of nucleoli is rDNA transcription, and it is essential for the successful development of ZGA^16^. Therefore, we investigated the nucleolar function-related gene expression (pre-rRNA, *Ubtf*, FBL, and B23). The results showed that those genes could be activated normally in the control along the embryonic development, but their expression was significantly lower in the *Dcaf13*^*♀*−/*♂*+^ group (Fig. 4b). H4K16Ac is a marker for silencing rDNA transcription^17^. We examined the H4K16Ac levels in late two-cell embryos by immunofluorescence, and we found that a remarkable increase occurred in *Dcaf13*^*♀*−/*♂*+^ embryos (Fig. 4c). These results revealed that the absence of maternal DCAF13 led to defects in nucleolus function and decrease in rRNA production at the two-cell stage.

To further assess the impact of DCAF13 maternal depletion on the ribosomal protein gene expression and rRNA transcription, we performed HPG staining to detect their translational activity, as they constitute the ribosome. After measuring the HPG staining intensity, as we predicted, a significant decrease was detected in *Dcaf13*^*♀*−/*♂*+^ late two-cell embryos (Fig. 4d).

These results indicated that zygotic genome activation in *Dcaf13*^*♀*−/*♂*+^ embryos was impaired. We also detected several representative zygotic genes that were highly transcribed in two-cell embryos^18^. Notably, all these genes were downregulated in *Dcaf13*^*♀*−/*♂*+^ two-cell embryos (Fig. 4e). This indicated that maternal DCAF13 was required for extensive zygotic gene transcription and not only for translation-related genes.

## Discussion

In mouse embryos, after fertilization, the first major developmental transition is the MZT^9^. The development depends on maternal protein and RNA stored by oocytes before ZGA, which occurs at the one-cell stage^15^. The MZT is the opening that embryos take charge of gene expression to control cell differentiation and further development instead of maternal origin. After fertilization, decondensation and reorganization of chromatin in male and female gametes is a crucial step needed for embryonic development^19^. In addition, the factors contributing to nucleus reformation before ZGA are strictly of maternal origin^8^. In this study, we investigated the critical factor contributing to zygotic nucleus reformation, and we identified DCAF13, a maternal protein located in the nucleoli of growing oocyte that played an important maternal role in zygotic nucleus reformation and was required for ZGA. Although DCAF13 expression is low in zygote and 2-cell embryos, maternal deletion of DCAF13 in growing oocytes still causes developmental arrest at this stage. So we selected this interesting model to elucidate the function of maternal DCAF13 during the early development of the zygotes. We showed that maternal DCAF13 was a major regulator of the chromatin tightness during MZT, especially for ribosomal protein gene transcription (as shown from RNA-seq data) during development. We believe that the MZT defect caused by DCAF13 deletion in oocytes was originated from the growing stage. Even if we supplied exogenous DCAF13 in fully grown GV stage oocytes, there was little chance for us to observe a rescuing effect.

**Figure 5:**
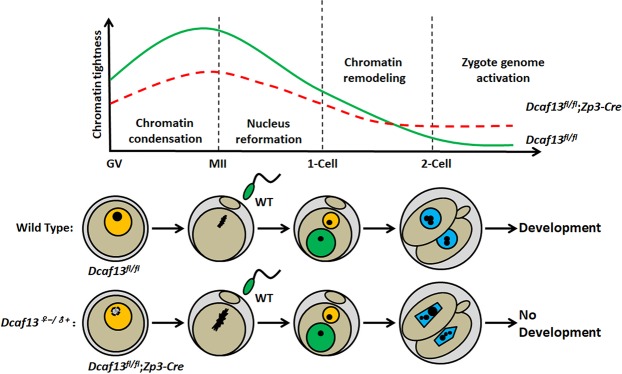
Schematic model for the role of maternal DCAF13 during the maternal-zygotic transition. In oocytes, the chromatin is condensed during NSN-SN transition, and is further condensed during meiotic maturation. After fertilization, chromatin structure turns to be extremely loose in pronuclei, leading to the whole genome started to be transcribed. However, the DCAF13-deleted oocytes have defects of chromatin tightening, as well as insufficient chromatin loosening after fertilization. Therefore, DCAF13 in growing oocyte is required for the compaction of chromatin during NSN-SN transition. Meanwhile, the correctly compacted genomic DNA in oocyte is also a prerequisite for embryonic chromatin loosening and nucleus reformation during MZT.

We showed that maternal DCAF13 was essential for chromatin tightness regulation during MZT. DCAF13 deletion in oocytes resulted in loose chromatin, and this influences nucleus reformation and caused chromatin condensation, which destroyed nucleus function. This prevented the development of embryos beyond the two-cell stage (Fig. 5). DCAF13-deleted oocytes possessed potential effects which were not vital for oocyte maturation but fatal for embryo development. In oocytes, the chromatin is condensed during NSN-SN transition, and is further condensed during meiotic maturation. After fertilization, the reverse process occurs. Chromatin structure turns to be extremely loose in pronuclei, leading to the whole genome started to be transcribed. The loosened chromatin structure is involved in their totipotency and high transcription activity. So the chromatin structure closely relates to transcription activity.

We observed defects of chromatin tightening (increased susceptibility of genomic DNA to DNase I digestion) in DCAF13-deleted oocytes, as well as insufficient chromatin loosening after fertilization. Based on the data presented in this manuscript, we proposed that DCAF13 in growing oocyte was required for the compaction of chromatin during NSN-SN transition. Meanwhile, the correctly compacted genomic DNA in oocyte is also a prerequisite for embryonic chromatin loosening and nucleus reformation during MZT. Maternal DCAF13 was the key factor in this process. DCAF13-dependent chromatin tightening in oocyte was required for appropriate transcription activation of early zygotic genes, particular those encoding rRNAs and ribosome proteins.

Reproductive aging is related to the decline in fertility, and endocrine functions draw public attention as women are globally delaying childbearing^20^. It is reported that the quality and developmental potentiality of oocytes are related to age, affecting the MZT and leading to embryonic development failure^21^. Moreover, a dataset of quantitative mass spectrometry of oocytes from young and aged female mice shows that DCAF13 protein is reduced in aged oocytes^22^. The reduction of DCAF13 influences oocyte competence, which is tightly connected to embryonic development^12^. Thus, our study provides an explanation of the reason why menopausal women are prone to miscarriage.

## Methods

### Mice

WT C57/B6 mice were obtained from the Shanghai SLAC laboratory, China. *Dcaf13*^*fl*/*fl*^ and *Zp3-Cre* mice exhibited a C57BL/6 background. The experimental procedures, and experimental protocols involving mice were approved by the Zhejiang University Institutional Animal Care and Research Committee (Approval # ZJU20170014), and mouse care and use were performed in accordance with the relevant guidelines and regulations.

### Western blot analysis

Zygotes were lysed with SDS sample buffer (100 zygotes per sample) and heated for 5 min at 95 °C. Total zygotes proteins were separated by SDS-PAGE and electrophoretically transferred to PVDF membranes (Millipore, USA), followed by blocking in TBST containing 5% defatted milk (BD, USA) for 30 min. First, the membranes were incubated with primary antibodies overnight at 4 °C. Then, the membranes were washed in TBST, and incubated with a HRP-linked secondary antibody for 1 h at room temperature, followed by washing with TBST three times. Finally, bounding antibodies were detected using SuperSignal WestFemto maximum sensitivity substrate (Thermo Fisher, USA). The primary antibodies used and dilution factors are anti-DDB1 (Epitomics, #3821-1, 1:10000) and anti-FLAG antibodies (Sigma, #F3165, 1:3000). The secondary antibody was HRP-conjugated anti-rabbit/mouse IgG (Jackson ImmunoResearch Laboratories).

### Immunofluorescent microscopy

Oocytes or embryos were fixed in phosphate-buffered saline-buffered 4% paraformaldehyde (PFA) and penetrated with 0.5% Triton X-100 (Sangon Biotech, China). They were blocked with 1% bovine serum albumin (BSA) (Sangon Biotech, China) in PBS. They were incubated with primary antibodies diluted in blocking solution. Next, they were washed in PBS, and labeled with secondary antibodies, counterstained with 40,6-diamidino-2-phenylindole (DAPI) (Sigma-Aldrich). Finally, they were mounted on glass slides using SlowFade Gold Antifade Reagent (Life Technologies). Imaging was performed on a Zeiss LSM710 confocal microscope. Single frame of each was scanned at the middle focal plane of their nucleus. Semi-quantitative analysis of the fluorescence signals was conducted using the NIH Image program ImageJ. Briefly, the pixel value/unit area was measured for the nucleus, and the value for the cytoplasm was subtracted as background. The value obtained was multiplied by the nuclear area to yield the total amount of fluorescence in the nucleus.

The primary antibodies used were anti-H4K16Ac (Abcam, ab109463, 1:400), anti-B23 (Abcam, ab10530, 1:1000). The secondary antibodies were Alexa Fluor 594-conjugated goat anti-rabbit/mouse IgG (Life Technology) and Alexa Fluor 488-conjugated goat anti-rabbit/mouse IgG (Jackson ImmunoResearch Laboratories, USA).

### *In vitro* transcription and preparation of mRNAs for microinjections

Expression vectors were linearized and subjected to phenol/chloroform extraction and ethanol precipitation. The linearized DNAs were *in vitro* transcribed using the SP6 message mMACHINE kit (Invitrogen, AM1340). Transcribed mRNAs were added with poly(A) tails using the mMACHINE kit (Invitrogen, AM1350) and were recovered by lithium chloride precipitation and resuspended in nuclease-free water.

### Superovulation and fertilization

Female mice at the pubertal stage (21–23 day old) were intraperitoneally injected with 5 IU of pregnant mare serum gonadotropin (PMSG, Ningbo Sansheng Pharmaceutical Co., ltd., P.R China). After 44 h, these mice were then injected with 5 IU of human chorionic gonadotropin (hCG, Ningbo Sansheng Pharmaceutical Co., ltd., P.R China). Immediately after hCG injection, female mice were mated with 10–12-week-old WT males that were known to be fertile. Successful coitus was confirmed by the presence of vaginal plugs the next morning. Preimplantation embryos were collected from oviducts at the indicated times after hCG injection.

### Microinjection of zygotes

For microinjection, mouse zygotes were harvested in M2 medium at 24 h after hCG injection. Microinjections were performed using an Eppendorf transferman NK2 micromanipulator. Each zygote was microinjected with approximately 5 to 10 pL samples. Microinjected zygotes were then washed with M2 and cultured in prewarmed KSOM medium at 37 °C with 5% CO_2_.

### TUNEL assay for detection of DNase I sensitivity

*Dcaf13*^*fl*/*fl*^ and *Dcaf13*^*fl*/*fl*^;*Zp3-Cre* GV oocytes or embryos were collected as described above and pre-extracted immediately in ice-cold solution (50 mM NaCl, 3 mM MgCl_2_, 0.5% Triton X-100, and 300 mM sucrose in 25 mM 4-(2-hydroxyethyl)-1-piperazineethanesulfonic acid (HEPES), pH 7.4) for 5 min. The oocytes were incubated with different concentrations of DNase 1 (NEB) for 5 min at 37 °C in the same buffer without Triton X-100 and fixed for 10 min in 2% PFA/PBS at room temperature. TUNEL assay was performed using Click-iT TUNEL Alexa Fluor Imaging Assay (Life Technologies, C10245) according to the manufacturer’s instructions^23^.

### 5-Ethynyl uridine (EU) staining and homopropargylglycine (HPG) staining

For EU staining, oocytes were incubated for 1 h with 100 mM EU from the Click-iT RNA Alexa Fluor 488 Imaging kit (Thermo Fisher Scientific). Oocytes were then fixed using 4% PFA/PBS for 15 min and permeabilized with 0.25% Triton/PBS for 15 min. The Click-It reaction was performed according to the manufacturer’s instructions. Oocytes were then counterstained with DAPI for 15 min before the slides were mounted with SlowFade Gold Antifade Reagent and imaged on a Zeiss LSM 710 at 63X. EU signal intensity was calculated using ImageJ^24^. The synthesis of protein was measured using Click-iT HPG Alexa Fluor Protein Synthesis Assay kits (Thermo Fisher Scientific) according the manufacturer’s instructions. Oocytes were treated with the Click-iT HPG reagent and then fixed with 4% PFA. Oocytes were permeated with 0.5% Triton X-100, and Alexa Fluor 488 was then conjugated to the protein using click chemistry. Fluorescence signal was detected using a Zeiss LSM 710 microscope and quantified using ImageJ.

### RNA-seq library preparation

Oocytes and embryos were collected from indicated genotypes (10 oocytes or embryos per sample). Each sample was added with 4 μl lysis buffer (0.2% Triton X-100, RNase inhibitor, dNTPs, oligo-dT primers and 100 pg *mCherry* mRNA spike-in) and immediately processed into cDNA using Smart-seq2 as described previously^25^. Sequencing libraries were constructed from 500 pg of pre-amplified cDNA using a DNA library preparation kit (TruePrep DNA Library Prep Kit V2 for Illumina, Vazyme) which based on Tn5 transposase. Barcoded libraries were pooled at equimolar ratios and sequenced on illumina platform with 150 bp pair-end reads.

### RNA-seq data analysis

RNA-seq was performed with biological replicates for all samples. RNA-seq reads were sequenced on the Illumina HiSeq platform as paired end 150-base reads. Raw reads were trimmed with Trimmomatic-0.36 to 50 bp and mapped to the mouse genome (mm9) with TopHat (v2.0.11). The mapped reads were subsequently assembled into transcripts guided by reference annotation (University of California at Santa Cruz [UCSC] gene models) with Cufflinks version 2.2.1. The expression level of each transcript was quantified with normalized FPKM based on the FPKM of exogenous *mCherry*. Differentially expressed genes were identified by asking for a fold change >10 with FPKM >1 in at least one sample. GO analysis for enrichment of GEGs (differentially expressed genes) was determined using the Database for Annotation, Visualization and Integrated Discovery (DAVID).

Statistical analyses were implemented with R (http://www.rproject.org). A Spearman’s R coefficient was calculated using the cor function with default parameters and complete method was used to cluster the genes.

### RNA isolation and reverse transcription polymerase chain reaction (RT-PCR)

Total RNA was extracted using RNeasy Mini kit (Qiagen, 74106). RT-PCR analysis was performed using the Power SYBR Green PCR Master Mix (Applied Biosystems, Life Technologies) and an Applied Biosystems 7500 Real-Time PCR System. Relative mRNA levels were normalized to the levels of exogenous *mCherry* mRNA (external control). The final concentration of *mCherry* in sample was 0.001 ng/µL. The relative transcript levels of samples were compared to the control, and the fold-changes were shown. Each experiment was conducted with three biological replicates.

### Statistical analysis

Results were expressed as means ± standard error of the mean (SEM). Most experiments included at least three independent samples and were repeated at least three times. Results for two experimental groups were compared by two-tailed unpaired Student’s *t*-tests. Statistically significant values of *p* < 0.05, *p* < 0.01, and *p* < 0.001 by two-tailed Student’s *t*-test were indicated by asterisks (*), (**), and (***) respectively. “ns” indicates non-significant.

The box and whiskers graphs in the manuscript were generated by Graphpad 6.0 (Graphpad Software Inc. USA). The box extended from the 25^th^ to 75^th^ percentiles. The whiskers went down to the smallest value and up to the largest. We have added more details about data processing in the method part.

## Supplementary information

Supplemental information

## Data Availability

The dataset generated and/or analysed during the current study is available from the corresponding author on reasonable request.
