# Supplementary material for: Maternal DCAF13 Regulates Chromatin Tightness to Contribute to Embryonic Development
Source: Sci Rep. 2019 Apr 18;9:6278. doi: 10.1038/s41598-019-42179-w (PMC6472424; doi:10.1038/s41598-019-42179-w)
Supplement: Supplementary file 1 — Supplemental information [file 41598_2019_42179_MOESM1_ESM.pdf]

# Maternal DCAF13 Regulates Chromatin Tightness to Contribute to Embryonic Development

**Yang Liu[1#], Long-Wen Zhao[2#], Jingling Shen[1], Heng-Yu Fan[2], Yan Jin[1]***

[1] Laboratory of Medical Genetics, Harbin Medical University, Harbin, China.

[2]Life Sciences Institute, Zhejiang University, Hangzhou 310058, China

[#]These authors contributed equally to this work.

***Corresponding authors:**

Dr. Yan Jin, Laboratory of Medical Genetics, Harbin Medical University, Harbin, China. Tel: 86-451-86674798; E-mail: jinyan@ems.hrbmu.edu.cn

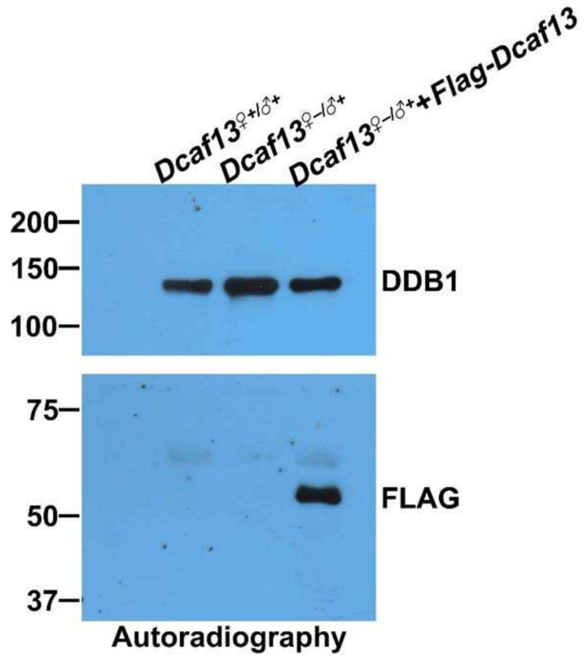


**Supplementary Figure S1: Uncropped scans of the Western blot results in Fig. 2H.**

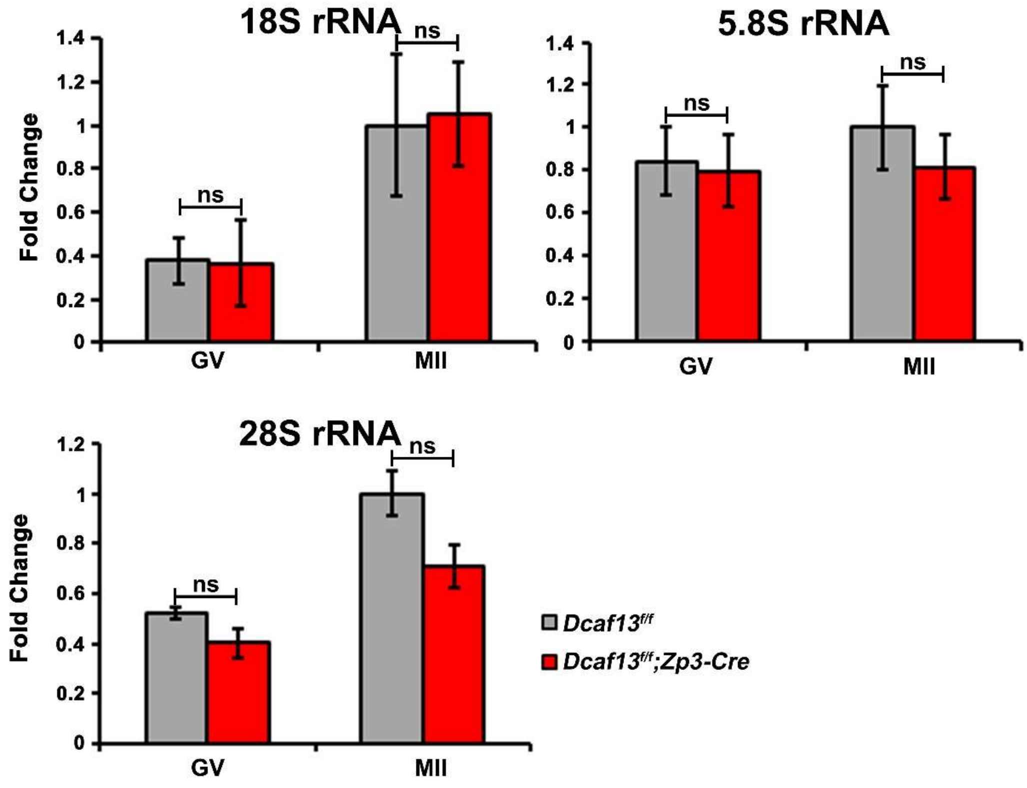


**Supplementary Figure S2: Graphical representation of the mean expression level ± standard error of the mean (SEM) by reverse transcription polymerase chain reaction (RT-PCR) for several ribosomal RNAs in *Dcaf13*$^{fl/fl}$ (in gray) and *Dcaf13*$^{fl/fl}$*;Zp3-Cre* (in red) oocytes.** ns: statistically nonsignificant ( $p > 0.05$).
